# Partner Influence in Diet and Exercise Behaviors: Testing Behavior Modeling, Social Control, and Normative Body Size

**DOI:** 10.1371/journal.pone.0169193

**Published:** 2016-12-29

**Authors:** Brea Perry, Gabriele Ciciurkaite, Christy Freadreacea Brady, Justin Garcia

**Affiliations:** 1 Department of Sociology, Indiana University, Bloomington, Indiana, United States of America; 2 Indiana University Network Science Institute, Indiana University, Bloomington, Indiana, United States of America; 3 Department of Sociology, Social Work, and Anthropology, Utah State University, Logan, Utah, United States of America; 4 Department of Sociology, University of Kentucky, Lexington, Kentucky, United States of America; 5 Department of Gender Studies, Indiana University, Bloomington, Indiana, United States of America; 6 The Kinsey Institute for Research in Sex, Gender, and Reproduction, Indiana University, Bloomington, Indiana, United States of America; TNO, NETHERLANDS

## Abstract

Previous research has documented social contagion in obesity and related health behaviors, but less is known about the social processes underlying these patterns. Focusing on married or cohabitating couples, we simultaneously explore three potential social mechanisms influencing obesity: normative body size, social control, and behavior modeling. We analyze the association between partner characteristics and the obesity-related health behaviors of focal respondents, comparing the effects of partners’ body type, partners’ attempts to manage respondents’ eating behaviors, and partners’ own health behaviors on respondents’ health behaviors (physical activity, fruit and vegetable consumption, and fast food consumption). Data on 215 partners are extracted from a larger study of social mechanisms of obesity in family and community contexts conducted in 2011 in the United States. Negative binomial regression models indicate that partner behavior is significantly related to respondent behavior (p < .001), net of controls. These results are suggestive of a behavior modeling mechanism in obesity-related patterns of consumption and physical activity. In contrast, we find little support for the influence of normative body size or partner social control in this sample, though generalizations about the relevance of these processes may be inappropriate. These results underscore the importance of policies and interventions that target dyads and social groups, suggesting that adoption of exercise or diet modifications in one individual is likely to spread to others, creating a social environment characterized by mutual reinforcement of healthy behavior.

## Introduction

Researchers have recently begun to explore whether social networks and relationships can be leveraged to help individuals achieve healthy lifestyles, examining social contagion in norms, behaviors, and outcomes [1]. This approach finds support in research conducted by Christakis and Fowler [2], which demonstrated that individuals’ weight losses and gains affected the weight of people who were associated directly or indirectly in their social networks. These relationships are strongest among married and romantic partners. Having an obese spouse increases one’s own risk for obesity by nearly forty percent [3, 4]. Understanding the social barriers to maintaining a healthy weight in current social and economic environments is paramount, as the goal of sustained weight management continues to be a challenging U.S. health priority [5].

Despite substantial research documenting the social contagion or convergence effect in obesity, the mechanisms underlying these patterns are poorly understood and rarely specified. At least three potential pathways have been proposed: First, social interaction may foster shared norms about ideal or acceptable body size, with greater tolerance of larger bodies leading to less effort toward weight control [6]. Second, individuals may exert social control over friends and family members, making explicit efforts to change others’ health behaviors to conform to their own behaviors or values [7]. Third, network effects in obesity may operate through behavior modeling, wherein people observe and adopt the behaviors of those around them, whether healthy or unhealthy [8].

Existing empirical research largely supports the role of shared body size norms in the social convergence of obesity [9]. The key idea is that the body types of people with whom a person interacts regularly influence one’s own ideas about ideal body type, and subsequently shape obesity-related behavior and outcomes. For example, Maximova and colleagues [6] found that overweight children and adolescents with obese parents and schoolmates were less likely to classify themselves as overweight than those with less exposure to obesity. Likewise, results from a large cross-national study [10] suggest that relative body mass index (i.e., how overweight one is in comparison to peers) is a more important indicator of self-perceptions of overweight than absolute body mass index. In contrast, Hruschka and colleagues [1] recently found that shared body size norms explain only a small amount of the social clustering of obesity. However, these authors focused only on women, and were not able to examine spousal/romantic partnerships. Research by Markey and Markey [11, 12] focusing explicitly on romantic partnerships found that ideal body size was not related one’s partner’s body size, but perceptions of one’s own actual body size were. Larger individuals with thinner partners were more likely to classify themselves as overweight compared to those with larger partners.

With respect to the role of social control in obesity convergence, existing studies have identified a relationship between network members’ attempts to regulate health behavior related to obesity or diet (e.g., exercise frequency, fat or salt content of foods) and increases in health-enhancing behavior [13, 14]. Romantic partners, in particular, can serve as powerful facilitators or obstructionists of healthy weight. They may act as agents of social control, encouraging healthy behaviors or discouraging unhealthy ones through coercion, bargaining, or restriction (e.g., purchasing only healthy foods for the household; [15]). For example, Markey, Gomel, & Markey [16] studied eating regulation in the context of heterosexual couples. They found that over half of the men and women in their sample reported attempts to regulate their partner’s eating behavior, and that this was more likely when the partner was overweight. Additionally, this study demonstrated that exertion of social control by partners was associated with greater concern about one’s weight and, among men, adoption of healthier behaviors. Other research by August and co-authors [17] provides a more complex picture of the outcomes of social control among couples. In two separate studies of individuals with Type-II diabetes and heterosexual college students, they found that behavioral and emotional outcomes of partner social control are mixed, influencing positive behavior change under some conditions, but also provoking hostility, behavioral resistance, and concealment of negative health behaviors in others [17–19].

Behavior modeling has also been suggested as a potential mechanism of obesity convergence in dyads, with strong support in the public health literature [2]. For instance, numerous studies have identified convergence of behavior among adolescents and their peers and family members with respect to physical activity and dietary habits [20–23]. Less research has been conducted on the concordance of health behaviors among coupled individuals, but existing studies have found evidence for correlation of eating behavior and nutritional intake [24, 25], as well as exercise patterns [26]. Moreover, research suggests that new couples tend to simultaneously develop healthy changes in eating behaviors (e.g., consuming more produce, reducing fat intake, and avoiding fast food; [27]), and that weight loss interventions delivered to one spouse have beneficial effects on the other. For example, Gorin and colleagues [28] found that untreated spouses of individuals participating in a weight loss intervention consumed fewer calories and fat compared to baseline measurement, lost significant weight, and also indicated decreases in the presence of high-fat foods in the home environment.

Despite the importance of existing research on social network effects in obesity, the specific mechanisms underlying this pattern are infrequently assessed. In addition, the majority of studies have focused on peer networks or inter-generational relationships, while partner convergence in obesity-related behaviors has received considerably less attention. Finally, to our knowledge, no study has directly compared the influence of social norms, social control, and behavior modeling on diet and exercise behaviors using one dataset. Exploring multiple alternative hypotheses simultaneously is critical for identifying the true effects of potentially correlated mechanisms, and adjudicating between them. This approach can also save resources by facilitating policy and programmatic decisions that focus on mechanisms that produce the largest effects on health outcomes.

The current paper addresses these gaps in our understanding of social convergence in obesity and related health behaviors through an analysis of partners’ characteristics and behaviors. Specifically, three complimentary processes are examined using measures designed for this purpose: normative body size, social control, and behavior modeling. First, we establish whether there is evidence of body congruence in partner dyads by examining relationships between partner and respondent body mass index (BMI) and body size. Then, we compare the effects of partner body type (normative body size), partner attempts to manage eating behavior (social control), and partner health behaviors (behavior modeling) on respondent health behaviors (physical activity, fruit and vegetable consumption, and fast food consumption).

## Methods

We analyzed data on 401 families from the Networks and Obesity: Relationships and Mechanisms Study (NORMS), fielded in 2011 to examine the impact of obesogenic relationships, networks, and community contexts on individual behaviors and outcomes. In addition to the data used in the present analysis, the survey contains information about interactions between parents and children around food choice, grocery shopping priorities and constraints, and children’s obesity-related behaviors and outcomes. All data collection activities were conducted with the approval and under the supervision of the University of Kentucky Institutional Review Board (Protocol #10-0396-P4S). Information about the study was provided via an IRB-approved informed consent form, which all participants signed. NORMS data were obtained via self-report surveys of adults with dependent children. The only inclusion criterion for participation in the study was being the parent or primary caregiver of a child between the ages of 2 and 17 years old.

Participants were recruited across ten sites (e.g., child care centers, schools, churches) in one urban area, Lexington, KY, USA, using deliberate sampling for heterogeneity. The goal of this strategy is to sample participants from a wide range of settings or social groups to achieve variation in characteristics that are expected to influence research findings [29]. This form of non-probability sampling is an appropriate alternative to random sampling when it is difficult to identify members of a population, or when a project has limited resources [30], and is regarded as stronger than convenience sampling or snowball sampling in instances where random sampling is not feasible. When using deliberate sampling for heterogeneity, there should be at least two subsamples chosen to represent the opposite ends of a continuum of potentially relevant social, economic, or other attributes [29]. In the current research, study sites were chosen to maximize the racial and socioeconomic heterogeneity of the sample. Specifically, we recruited participants from child care facilities, academic programs, and churches which 1) primarily serve families with the highest or lowest socioeconomic backgrounds (i.e., education and income) in the metro area; and 2) those serving a majority of white families or a majority of African American families. Consequently, the sample demographic characteristics are not nationally representative by design, and inferential statistics should be interpreted with caution.

In the 10 sampling sites, all parents were first informed about the study through emails and/or personal contact from directors of the organization sites and via fliers. Then, surveys were distributed to all families and were completed and returned during parent meetings at the facility, or were completed at home and returned via mail (self-addressed, postage-paid envelopes) or using locked drop boxes in the various facilities. Across all of these sites, the response rate was 65%.

A total of 401 respondents completed the NORMS survey. Of these, 246 respondents were currently living with a partner or spouse, and this subsample was used to study partner influence on obesity-related health behaviors. A total of 31 cases (13%) were dropped from the partnered sample using listwise deletion due to missing data on one or more variables in this analysis. Dropped cases did not differ from cases included in the analysis on any independent or dependent variables with the exception of race. Namely, about 46% of cases dropped due to missing data identified as white, while 54% identified as a member of a racial or ethnic minority group (*p* < .05). Consequently, minority groups might have been slightly underrepresented. The final analysis sample included 215 participants living with a partner and children in the home.

### Measures

Three types of variables were included in this analysis: Respondent health behaviors, sociodemographic and BMI controls, and mechanisms of partner influence. The main dependent variables were three measures of obesity-related health behaviors. Physical activity was reported as number of days per week, on average, the respondent “plays actively or exercises for 20 or more minutes.” Produce consumption was the sum of number of times per day the respondent reported eating “fresh or canned fruit” and “vegetables, including salad.” Fast food consumption was the number of times per month the respondent ate fast food (e.g., McDonalds). We also initially examined soft drink consumption as a dependent variable. Observed patterns were identical to the other dependent variables (i.e., findings provided evidence of behavior modeling, but not social control or normative body size). However, we did not feel that these analyses added much to the weight of evidence given the three other outcomes. Results are available on request.

One additional dependent variable was employed to test the normative body size mechanism. Namely, respondents were asked to choose their ideal body size using the Collins Adult Female (or Male) Scale [31] comprised of a series of 7 drawn figures, ranging from underweight to very obese. The range was 1–7, with higher values depicting heavier bodies.

Sociodemographic information was modeled as a series of independent control variables. These included gender (1 = women; 0 = men) and race (1 = white; 0 = nonwhite). Racial and ethnic minorities were collapsed into one category due to insufficient cell sizes (i.e., 50 of 64 were Black). Education was measured in years of schooling, and marital status was indexed using a binary variable (1 = currently married; 0 = cohabitating but unmarried).

All models included respondent BMI to control for the potential confounding effects of overweight and obesity in the relationship between health behavior and partner influence. BMI was calculated using respondent-reported height and weight. Because BMI has been widely criticized [32, 33], other variables were included in models to test sensitivity to alternative measures of overweight and obesity: current body size perceptions using the Collins [31] Scale, self-reported weight category (i.e., “How would you describe your weight”), and self-reported health. We also reran analyses using a series of dummy variables representing BMI categories (e.g., normal weight, obese, etc.). None of these other measures performed as well as continuous BMI, nor differently influenced the coefficients of interest. Also, they were highly correlated (e.g., BMI and body size perceptions were correlated at 0.78; p < .001).

Key independent variables operationalized three mechanisms of partner influence in health behaviors: normative body size, social control, and behavior modeling. Normative body size was tested using a measure of the respondent’s perception of the partner’s body size, as depicted on the Collins Scale [31]. Alternative measures of partner overweight or obesity were employed, but these did not change the substantive conclusions. These included partner BMI and respondent’s perception of the partner’s weight category (e.g., “How would you describe your partner’s weight?”). Partner body size was ultimately retained because it best fit the theory of normative body size. It ranged from 1 to 7, with higher values signifying heavier bodies.

Partner social control was indexed using a single item. It asked, “To what extent does your partner or spouse attempt to manage or control what or how much (you) eat?” Response categories were “very much,” “a fair amount,” “somewhat,” “not very much,” and “not at all.” This variable was recoded such that higher values indicated more social control. The operational fit between social control of eating behaviors and diet-related outcomes is clearly stronger than the fit with physical activity. However, we analyze the relationship between partner control of eating behaviors and exercise days for consistency, and because this measure can be conceptualized as a proxy for partner social control of health behaviors, more generally.

Behavior modeling was assessed using a series of items about partner health behaviors that mirrored those used as dependent variables. Namely, partner physical activity, produce consumption, and fast food consumption were included in the corresponding model of respondent behavior. These were coded identically to respondent behaviors.

### Analysis

To establish an empirical foundation of body congruence among couples, we first examined bivariate associations between respondent and partner BMI and body size using Pearson correlations. To test study hypotheses, multivariate negative binomial regression models were conducted using a sequential strategy. The key independent variables measuring normative body image, social control, and behavior modeling were x-standardized to make effects directly comparable, and to use overlapping confidence intervals as an indicator of significantly different effect sizes. Negative binomial regressions were used and incidence rate ratios (IRR) presented because these were count outcomes and the models were zero-inflated. All models included the following control variables: respondent gender, race, education (in years), marital status, and BMI. For each dependent variable (i.e., respondent health behavior), Model 1 included control variables plus partner body size, Model 2 included control variables plus partner social control, and Model 3 included control variables plus partner health behaviors. Finally, Model 4 (full model) included all variables from Models 1–3, including controls. To provide a more robust test of the normative body size hypothesis, we also computed an ordinal logistic regression model regressing respondent ideal body size on sociodemographic controls and partner body size. For all regressions, violations of model assumptions were examined and found not to be problematic.

Finally, because previous research has sometimes identified gender and marital status differences in the effects of social influence on health behavior [2, 17, 34, 35], we added gender*covariate and married*covariate interaction terms separately to each model. For marital status, the direction of the interaction was in the expected direction (i.e., social influence was stronger for married respondents compared to non-married), but did not achieve statistical significance. No discernable patterns were evident for the gender interactions, and they were also non-significant. It is possible that non-significance was due, in part, to the small number of male and unmarried respondents, so these null interactions should be interpreted with caution. The interaction terms were not included in the final models.

## Results

Basic descriptive statistics are presented in Table 1. About 81% of respondents were women and 19% were men. With respect to race/ethnicity, 70% of respondents were white, 23% were African American, 4% were Asian, 1% were Latino, and the remaining 2% identified as another race or multiracial. African Americans were overrepresented in the sample. The mean years of schooling was about 16, reflecting a disproportionately educated sample. However, the median household income was $62,500 USD—substantially lower than the national median of $85,087 USD for a married couple with two children [36]. Moreover, 8% of respondents in the sample fell below the federal poverty line. Finally, around 87% of couples in the sample were currently married. Among the unmarried 13% (n = 28) of the sample (all of whom had current partners), 29% were cohabitating (n = 8). In addition, 43% (n = 12) of the unmarried respondents had been married previously.

**Table 1:** Sample characteristics for partnered adults, NORMS (n = 215).

	% (*n*)	$\bar{\text{X}}(\text{s})$	Range
*Respondent socio-demographics*			
Female	81.40 (175)		
White	70.23 (151)		
Education (years)		16.43 (2.68)	10.00–22.00
Currently married	86.98 (187)		
*Respondent health and behavior*			
BMI		27.53 (6.66)	16.82–51.69
Ideal body size		3.67 (0.77)	2.00–7.00
Physical activity (days/week)		2.83 (1.88)	0.00–7.00
Produce consumption (times/day)		3.42 (1.67)	0.00–8.00
Fast food consumption (days/month)		4.98 (5.30)	0.00–30.00
*Partner health and behavior*			
BMI		28.85 (5.46)	18.24–47.46
Current body size		4.67 (1.14)	2.00–7.00
Social control of respondent’s diet		2.28 (1.28)	1.00–5.00
Physical activity (days/week)		3.04 (2.24)	0.00–7.00
Produce consumption (times/day)		3.06 (1.85)	0.00–8.00
Fast food consumption (times/month)		5.73 (5.81)	0.00–30.00

Descriptive statistics indicated that the sample was similar to the American population as a whole with respect to BMI (mean = 27.5, compared to 27.9 for the average adult American man and 28.2 for a woman; [37]). Mean exercise (2.83 times/week) in the sample may have met Department of Health and Human Services (DHHS) guidelines (i.e., a minimum of 150 minutes per week; [38]), but this is unknown because the duration of exercise was not measured. Mean fruit and vegetable consumption (3.42) was well below the DHHS recommended daily intake of 5 total fruit and vegetable servings per day [39]. In addition, there were significant correlations between respondent and partner BMI (r = 0.33, *p* < .001), and between respondent and partner body size, as measured on the Collins [31] picture scale (r = 0.26, *p* < .001). The observed body congruence supported the need for this analysis of mechanisms of social convergence in obesity-related health behaviors in marital and cohabitating partnerships. Additionally, respondent and partner physical activity, produce consumption, and fast food consumption were correlated at r = 0.43 (p < .001), r = 0.72 (p < .001), and r = 0.73 (p < .001), respectively.

Findings on the influence of partner body size, social control, and behavior on respondent physical activity are presented in Table 2. BMI was negatively associated with physical activity, as expected (*p* < .01), and women were predicted to exercise less frequently than men (*p* < .01). Also, while there was no significant effect of partner body size or social control of eating behavior, we did find that partners’ exercise habits were positively related to respondents’ level of physical activity (*p* < .001). Specifically, for each one standard deviation increase in times per week that a partner exercised, the frequency of respondent exercise increased by 13%, controlling for respondent BMI and other potential confounding factors (See Model 4). Moreover, the incidence rate ratio for standardized partner physical activity was significantly larger than the incidence rate ratios for partner body size (p < .05) and social control of eating behavior (p < .05).

**Table 2:** Negative binomial regression of respondent physical activity on partner body size, social regulation, and behavior (n = 215).

	Model 1	Model 2	Model 3	Model 4
	IRR (CI)	IRR (CI)	IRR (CI)	IRR (CI)
Female	0.80* (0.64–0.99)	0.82 (0.65–1.02)	0.75** (0.61–0.92)	0.76** (0.62–0.93)
White	1.19 (0.97–1.46)	1.25* (1.01–1.55)	1.14 (0.94–1.37)	1.16 (0.96–1.42)
Education (years)	0.99 (0.95–1.02)	0.99 (0.95–1.02)	0.99 (0.96–1.03)	0.99 (0.96–1.03)
Currently married	0.98 (0.75–1.30)	0.94 (0.71–1.24)	1.03 (0.80–1.32)	1.01 (0.78–1.30)
BMI	0.98** (0.97–0.995)	0.98** (0.96–0.99)	0.98* (0.97–0.996)	0.98** (0.97–0.996)
Partner body size (std)	0.96 (0.87–1.05)			1.03 (0.94–1.13)
Partner social control (std)		1.06 (0.96–1.17)		1.03 (0.94–1.13)
Partner physical activity (std)			1.32*** (1.22–1.44)	1.32*** (1.22–1.44)
*Pseudo-R*^*2*^	0.02	0.02	0.07	0.07
*Chi-square*	14.66*	15.25*	57.79***	58.26***

Note: IRR = incidence rate ratio; CI = confidence interval; std = x-standardized;

*p < .05;

**p < .01;

***p < .001.

Results from regression models predicting produce consumption are presented in Table 3. In the full model (See Model 4), women (*p* < .05) were predicted to consume fruits and vegetables more frequently than men. With regard to the influence of normative body size, we found that as standardized partner body size increased, produce consumption decreased, on average (Model 1; *p* < .05). Additionally, partners’ produce consumption was strongly related to respondent’s eating behavior, with each one standard deviation increase in the number of fruits or vegetables consumed by partners being associated with a 20% increase in respondent fruit or vegetable consumption (Model 4; *p* < .001). The effects of partner body size on respondent fruit and vegetable consumption became nonsignificant in Model 4, when partner produce consumption was included. An analysis of bivariate association suggests substantial correlation of partner produce consumption with partner body size (r = -0.28, *p* < .001). Thus, results in Model I likely reflected the confounding effect of behavior modeling rather than the influence of normative body image or social control. Also, as shown in Model 4, the incidence rate ratio for standardized partner produce consumption was significantly larger than the incidence rate ratios for partner body size (p < .01) and social control of eating behavior (p < .01).

**Table 3:** Negative binomial regression of respondent produce consumption on partner body size, social regulation, and behavior (n = 215).

	Model 1	Model 2	Model 3	Model 4
	IRR (CI)	IRR (CI)	IRR (CI)	IRR (CI)
Female	1.26* (1.03–1.55)	1.31* (1.06–1.61)	1.30* (1.06–1.60)	1.29* (1.05–1.59)
White	0.92 (0.78–1.08)	0.98 (0.82–1.17)	0.97 (0.82–1.15)	0.97 (0.81–1.15)
Education (years)	1.01 (0.98–1.04)	1.01 (0.98–1.04)	1.03 (1.00–1.06)	1.03 (1.00–1.06)
Currently married	0.90 (0.72–1.12)	0.85 (0.68–1.06)	0.86 (0.69–1.07)	0.86 (0.69–1.08)
BMI	0.99 (0.98–1.01)	0.99 (0.98–1.00)	0.99 (0.98–1.00)	0.99 (0.98–1.01)
Partner body size (std)	0.92* (0.87–0.99)			1.01 (0.94–1.08)
Partner social control (std)		1.08 (0.99–1.17)		0.99 (0.92–1.05)
Partner produce consumption (std)			1.38*** (1.29–1.47)	1.39*** (1.29–1.49)
*Pseudo-R*^*2*^	0.02	0.02	0.12	0.12
*Chi-square*	14.52*	12.54	94.93***	95.24***

Note: IRR = incidence rate ratio; CI = confidence interval; std = x-standardized;

*p < .05;

***p < .001

Table 4 depicts results on fast food consumption. These revealed that BMI was positively associated with frequency of eating fast food (*p* < .01). In Model 3, standardized partner fast food consumption was positively correlated with respondent fast food consumption (IRR = 1.79, *p* < 0.001), all else equal, and this result was robust to the addition of all covariates in Model 4. Partner body size also achieved significance in the full model, with each one-standard deviation increase in partner body size on the Collins scale [31] being associated with an 11% increase in the number of respondent visits to fast food restaurants per month (*p* < .05). However, the incidence rate ratio for standardized partner fast food consumption was significantly larger than the incidence rate ratios for partner body size (p < .05) and social control of eating behavior (p < .01).

**Table 4:** Negative binomial regression of respondent fast food consumption on partner body size, social regulation, and behavior (n = 215).

	Model 1	Model 2	Model 3	Model 4
	IRR (CI)	IRR (CI)	IRR (CI)	IRR (CI)
Female	0.88 (0.63–1.22)	0.86 (0.62–1.20)	0.93 (0.73–1.19)	0.93 (0.73–1.19)
White	1.09 (0.83–1.45)	1.03 (0.76–1.39)	0.88 (0.71–1.09)	0.91 (0.72–1.14)
Education (years)	0.92** (0.88–0.97)	0.92*** (0.88–0.97)	0.96 (0.93–1.00)	0.96 (0.93–1.00)
Currently married	1.01 (0.67–1.50)	1.07 (0.71–1.62)	1.31 (0.97–1.78)	1.27 (0.93–1.73)
BMI	1.02* (1.00–1.04)	1.03** (1.01–1.05)	1.02*** (1.01–1.04)	1.02** (1.01–1.03)
Partner body size (std)	1.06 (0.93–1.21)			1.11* (1.004–1.22)
Partner social control (std)		0.94 (0.82–1.08)		1.03 (0.93–1.14)
Partner fast food consumption (std)			1.79*** (1.63–1.97)	1.80*** (1.64–1.98)
*Pseudo-R*^*2*^	0.02	0.02	0.13	0.14
*Chi-square*	21.13**	21.06**	151.32***	155.65***

Note: IRR = incidence rate ratio; CI = confidence interval; std = x-standardized;

*p < .05;

**p < .01;

***p < .001.

To provide an additional test of the normative body size hypothesis, we also tested the effects of partner body size and partner BMI on respondents’ ideal body size, controlling for respondent sociodemographic information and BMI. Neither partner body size (OR = 1.16, *p* = 0.28) nor partner BMI (OR = 0.98, *p* = 0.39) were significantly associated with respondent ideal body size, consistent with prior research [11, 12], providing strong support for rejecting the normative body image hypothesis. These results were not presented in tables due to non-significance.

## Discussion

Although the clustering of obesity in social networks has been well documented, little previous research has attempted to uncover the mechanisms through which social convergence operates in dyads. In the current study, we first demonstrated correlation between partner and respondent BMI and current body size, consistent with previous research on obesity social contagion [3, 4]. Taking this as a starting point, we then simultaneously tested three different mechanisms through which partner convergence in obesity may occur—shared body size norms, social control, and behavior modeling. We focused specifically on the role of health behaviors that are strongly predictive of obesity [40, 41], determining whether respondent physical activity and diet are influenced by partner body size, partner social control of behavior, and/or partner behavior. Our results provide evidence that is suggestive of a behavior modeling mechanism in obesity-related health behaviors among married and cohabitating partners. In contrast, we found almost no support for the influence of normative body size on the physical activity and frequency of consumption of produce and fast food among coupled individuals. In fact, partner body size was not significantly related to respondent ideal body size. Partners’ attempts to control respondents’ food consumption also did not emerge as a significant predictor of health behaviors, including diet.

Our findings extend existing research on social convergence in obesity by directly comparing potential mechanisms of network effects. Across four different behavioral outcomes, partner behavior emerged as a significant predictor, and in all cases this effect was significantly larger in magnitude than the incidence rate ratios for normative body image and social control. Given the amount of time spent in the presence of one’s partner, it is logical that modeling of health behaviors would occur in the context of marital and cohabitating relationships. Partnership provides ample opportunity for observing the behavior of the other person (e.g., partners often eat together), and for witnessing the consequences of that behavior—whether it be the immediate taste reward of negative behaviors like fast food consumption, or the long-term benefits of regular physical activity on health and body size. Moreover, having a strong attachment to the other person may heighten identification with his or her experiences and the motivation to conform behaviorally. Indeed, previous work on close relationships has demonstrated that people tend to integrate their sense of self with close others, and partners often even identify as an overlapping self [42]. The powerful influence of behavior modeling among close associates may explain why obesity convergence is stronger among spouses relative to other types of social ties [2, 24].

Our research has important limitations. First, measures of partner body size, social control, and behavior were reported by respondents rather than observed directly or reported by partners (i.e., proxy reporting). This limitation is offset to some degree by the argument that respondents’ perceptions of partner characteristics should be more influential than actual ones [43]. In addition, research suggests that proxy reporting is most accurate when the subject is a close tie, and particularly a spouse or cohabitating partner [44]. The accuracy of proxy reports is also optimized when the information is behavioral (i.e., observable) rather than attitudinal [45, 46], reducing concerns about the reliability of partner behavioral information. Second, because our sample is nearly 80% women, our findings may not extend to men. The gender composition of the sample may explain why we did not observe significant effects of social control, which have sometimes been reported exclusively for men [16]. Third, the single-item measure of social control that was available in the dataset is not optimal since previous research has found that some forms of control are associated with positive behavioral outcomes and others are not [17–19]. Future research should compare these social mechanisms of social influence using multiple and multidimensional indices. Fourth, our measure of BMI is self-reported, which may have led to systematic underreporting by heavier individuals. Fifth, we have no measure of the length of the relationship. Because convergence in health behaviors may be a process that occurs slowly over time, potentially becoming weaker or stronger as a relationship progresses, future research should consider this and other relationship factors.

Finally, our data are cross-sectional, limiting our ability to infer causality or establish directionality in the associations between respondent and partner health behaviors. It is entirely possible, for example, that respondent health behaviors are driving partner behaviors rather than the reverse, though this possibility does not threaten the face validity of a behavior modeling explanation. Moreover, given the cross-sectional nature of the data, we cannot determine the extent to which assortative mating (i.e., preferentially choosing a partner that is behaviorally homophilous to oneself) is driving the results that we attribute to behavior modeling. However, these models do control for race, socioeconomic status, and BMI—all of which are probably more likely to drive assortative mating, and therefore confound the behavior modeling explanation, than behavioral homophily in diet and exercise [47]. Moreover, given existing longitudinal research suggesting that the health behaviors of partners do converge subsequent to coupling, it is highly unlikely that all or even most of the behavioral similarity observed here is due to assortative mating [26, 27].

## Conclusions

Despite the aforementioned limitations, this study offers important insights into potential mechanisms underlying social influence in obesity. Our findings suggest that the social convergence of body weight may operate in part through the observation and adoption of health behaviors that contribute to weight control or obesity. We focus on partner convergence in behavior, which has received considerably less attention than peer or parental effects. These results lend support to weight loss or fitness interventions that target dyads and groups—including couples and families—suggesting that adoption of exercise or diet modifications in one individual may spread to proximate others, creating a social environment characterized by mutual reinforcement of healthy behavior. Research suggests that a person who makes a healthy behavior change has a larger impact on others’ positive health behaviors than one whose behavior is consistently healthy [48], underscoring the value of these kinds of group interventions. This also has implications for workplace weight control programs, with potential for extension to schools and other institutions in which regular social interaction occurs.

Additionally, our findings may have implications for public health initiatives. For example, prior research indicates that ideal BMI is largely determined by one’s own current BMI rather than by normative ideal body size [49]. In conjunction with our findings, this suggests that social interaction with people who are not overweight or obese is unlikely to affect one’s own ideal body type [11, 12], let alone behavior, and therefore may not be a viable explanation for the social convergence of obesity. Consequently, public health campaigns that seek to promote medical norms of ideal body size are not likely to be effective. Our results on social control may likewise shed light on the lack of observable relationships between paternalistic public health policies around weight control (e.g., taxation or partial bans on soft drinks; [50]) and population levels of overweight and obesity. Direct social control—whether interpersonal or structural—may not be the most effective mechanism of health behavior change, though additional research is needed to confirm this result. In all, our findings suggest that research which directly compares potential mechanisms of network effects in obesity is critical for saving resources and identifying efficient policy and programmatic initiatives by focusing on mechanisms that produce the largest effects on health outcomes.

## Supporting Information

S1 File NORMS Survey Instrument. (DOCX)
