# Supplementary material for: Partner Influence in Diet and Exercise Behaviors: Testing Behavior Modeling, Social Control, and Normative Body Size
Source: PLoS One. 2016 Dec 29;11(12):e0169193. doi: 10.1371/journal.pone.0169193 (PMC5199005; doi:10.1371/journal.pone.0169193)
Supplement: S1 File — (DOCX) [file pone.0169193.s001.docx]

**Networks and Obesity: Relationships and Mechanisms Study (NORMS)**

1. What is your race or ethnicity?

1 Caucasian/White 5 Asian or Asian American

2 American Indian or Alaska Native 6 Hispanic or Latino

3 Hawaiian or Other Pacific Islander 7 Two or more races/ethnicities

4 African American/Black

2. What is your current marital status?

1 Married 4 Divorced

2 Widowed 5 Separated

3 Single/Never been married 6 Cohabitating

3. What is your current employment status?

1 Employed full-time for wages 5 Employed part-time for wages

2 Out of work for more than 1 year 6 Out of work for less than 1 year

3 Homemaker 7 Student

4 Retired 8 Unable to work

4. Circle the highest level of education you completed:

1 Grades 1 through 8 (Elementary or middle school)

2 Grades 9 through 11 (Some high school)

3 Grade 12 or GED (High school graduate)

4 College 1 year to 3 years (Some college or technical school)

5 College 4 years (College graduate)

6 Master’s Degree

7 Doctoral / Professional Degree

5. Circle the highest level of education **your spouse/partner/significant other** completed:

1 Grades 1 through 8 (Elementary or middle school)

2 Grades 9 through 11 (Some high school)

3 Grade 12 or GED (High school graduate)

4 College 1 year to 3 years (Some college or technical school)

5 College 4 years (College graduate)

6 Master’s Degree

7 Doctoral / Professional Degree

6. Please tell me your approximate family/household income before taxes in 2010.

1 Less than $5,000 8 $45,001 - $60,000

2 $5,001 -$10,000 9 $60,001 - $75,000

3 $10,001 - $15,000 10 $75,001 - $100,000

4 $15,001 - $20,000 11 $100,001 - $125,000

5 $20,001 - $25,000 12 $125,001 - $150,000

6 $25,001 - $35,000 13 Greater than $150,000

7 $35,001 -$45,000

|  | **You** | **Partner or spouse** | **Child 1** | **Child 2** | **Child 3** | **Child 4** | **Child 5** | **Child 6** |
| --- | --- | --- | --- | --- | --- | --- | --- | --- |
| Age |  |  |  |  |  |  |  |  |
| Height |  |  |  |  |  |  |  |  |
| Weight |  |  |  |  |  |  |  |  |
| Sex | M F | M F | M F | M F | M F | M F | M F | M F |
| On an average day, how many times does each eat fresh or canned fruit? |  |  |  |  |  |  |  |  |
| On an average day, how many times does each eat vegetables, including salad? |  |  |  |  |  |  |  |  |
| How many times a month does each eat fast food (e.g., McDonalds)? |  |  |  |  |  |  |  |  |
| How many sodas per week do each drink? |  |  |  |  |  |  |  |  |
| How many times per week does each play actively or exercise for 20 or more minutes? |  |  |  |  |  |  |  |  |
| About how many hours does each person sit and watch television or play video games on an average day? |  |  |  |  |  |  |  |  |
| How many times a day does each brush their teeth? |  |  |  |  |  |  |  |  |
| How many cavities has this person had? |  |  |  |  |  |  |  |  |
|  | **You** | **Partner or spouse** | **Child 1** | **Child 2** | **Child 3** | **Child 4** | **Child 5** | **Child 6** |
| How would you rate each person’s overall health? Circle one:  1=Poor  2=Fair  3=Good  4=Very good  5=Excellent | 1  2  3  4  5 | 1  2  3  4  5 | 1  2  3  4  5 | 1  2  3  4  5 | 1  2  3  4  5 | 1  2  3  4  5 | 1  2  3  4  5 | 1  2  3  4  5 |
| How would you describe each person’s weight? Circle one:  1=Very underweight  2=Slightly underweight  3=About the right weight  4=Slightly overweight  5=Very overweight | 1  2  3  4  5 | 1  2  3  4  5 | 1  2  3  4  5 | 1  2  3  4  5 | 1  2  3  4  5 | 1  2  3  4  5 | 1  2  3  4  5 | 1  2  3  4  5 |
| To what extent do you worry that this person is or may become overweight?  1=Very much  2=A fair amount  3=Somewhat  4=Not very much  5=Not at all | 1  2  3  4  5 | 1  2  3  4  5 | 1  2  3  4  5 | 1  2  3  4  5 | 1  2  3  4  5 | 1  2  3  4  5 | 1  2  3  4  5 | 1  2  3  4  5 |
| To what extent does your partner/spouse worry that this person is or may become overweight?  1=Very much  2=A fair amount  3=Somewhat  4=Not very much  5=Not at all | 1  2  3  4  5 | 1  2  3  4  5 | 1  2  3  4  5 | 1  2  3  4  5 | 1  2  3  4  5 | 1  2  3  4  5 | 1  2  3  4  5 | 1  2  3  4  5 |
|  |  |  |  |  |  |  |  |  |
|  |  |  |  |  |  |  |  |  |
|  | **You** | **Partner or spouse** | **Child 1** | **Child 2** | **Child 3** | **Child 4** | **Child 5** | **Child 6** |
| To what extent do you attempt to manage or control what or how much this person eats?  1=Very much  2=A fair amount  3=Somewhat  4=Not very much  5=Not at all | 1  2  3  4  5 | 1  2  3  4  5 | 1  2  3  4  5 | 1  2  3  4  5 | 1  2  3  4  5 | 1  2  3  4  5 | 1  2  3  4  5 | 1  2  3  4  5 |
| To what extent does your partner or spouse attempt to manage or control what or how much this person eats?  1=Very much  2=A fair amount  3=Somewhat  4=Not very much  5=Not at all | 1  2  3  4  5 | 1  2  3  4  5 | 1  2  3  4  5 | 1  2  3  4  5 | 1  2  3  4  5 | 1  2  3  4  5 | 1  2  3  4  5 | 1  2  3  4  5 |
| Looking at the images of adults or children on the following page, circle the number that most closely resembles each person’s body shape now. | 1 5  2 6  3 7  4 | 1 5  2 6  3 7  4 | 1 5  2 6  3 7  4 | 1 5  2 6  3 7  4 | 1 5  2 6  3 7  4 | 1 5  2 6  3 7  4 | 1 5  2 6  3 7  4 | 1 5  2 6  3 7  4 |
| Looking at the images of adults or children on the following page, circle the number that most closely resembles each person’s ideal body shape, in your opinion. | 1 5  2 6  3 7  4 | 1 5  2 6  3 7  4 | 1 5  2 6  3 7  4 | 1 5  2 6  3 7  4 | 1 5  2 6  3 7  4 | 1 5  2 6  3 7  4 | 1 5  2 6  3 7  4 | 1 5  2 6  3 7  4 |


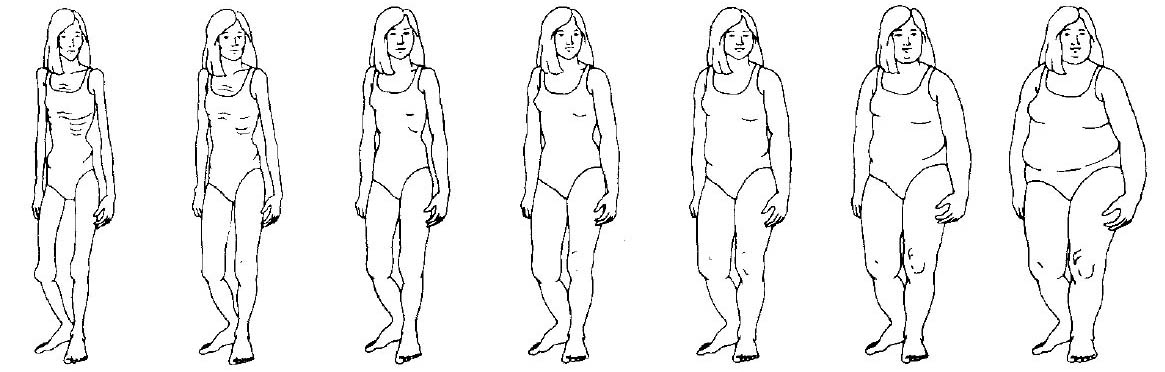


1 2 3 4 5 6 7


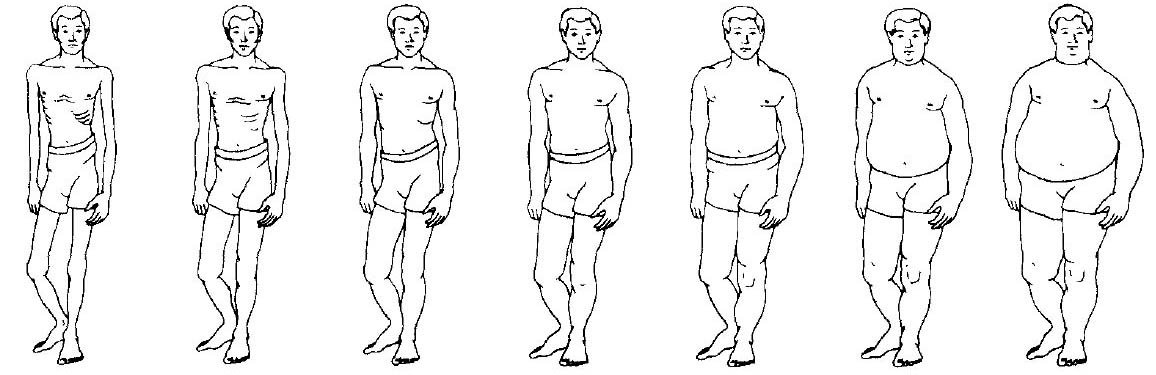


1 2 3 4 5 6 7


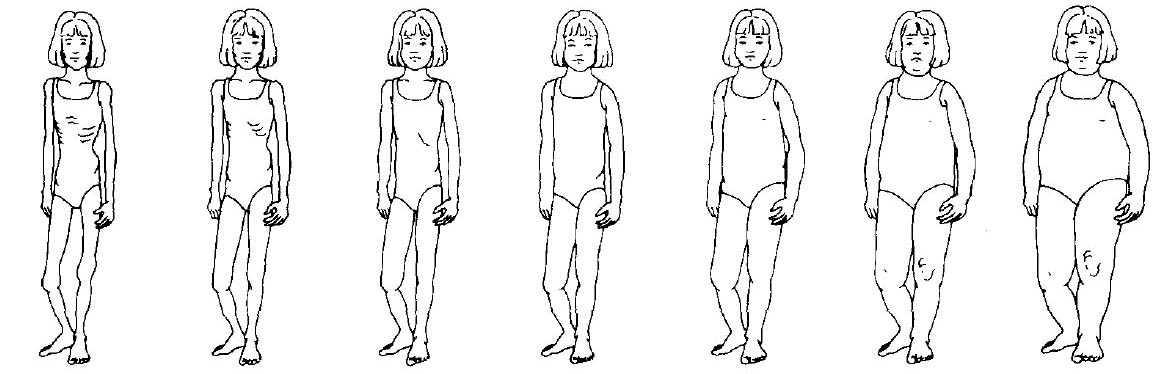


1 2 3 4 5 6 7


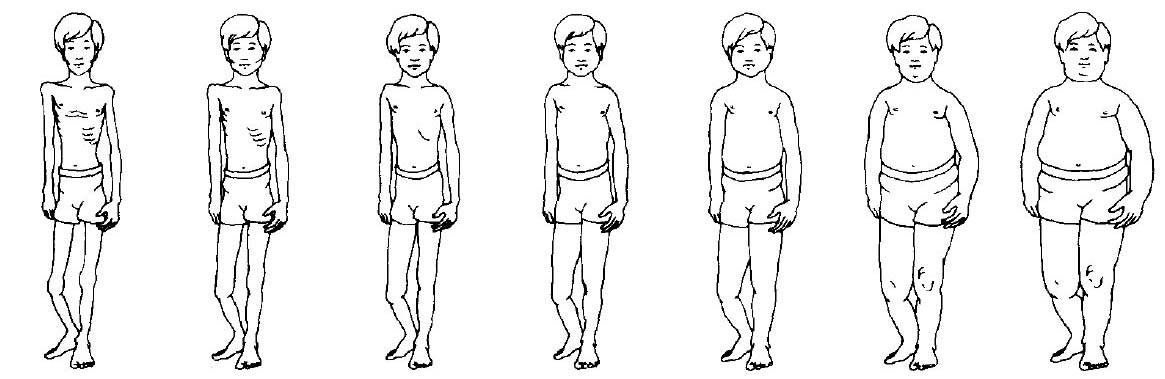


1 2 3 4 5 6 7
